# Decreased plasma levels of PDGF-BB, VEGF-A, and HIF-2α in preterm infants after ibuprofen treatment

**DOI:** 10.3389/fped.2022.919879

**Published:** 2022-07-26

**Authors:** Xuemei Huang, Dongshan Han, Yanfei Wei, Bingchun Lin, Dingyuan Zeng, Yu Zhang, Ba Wei, Zhifeng Huang, Xueyu Chen, Chuanzhong Yang

**Affiliations:** ^1^Department of Neonatology, Shenzhen Maternity and Child Healthcare Hospital, The First School of Clinical Medicine, Southern Medical University, Shenzhen, China; ^2^Department of Neonatology, Liuzhou Maternity and Child Healthcare Hospital, Affiliated Maternity Hospital and Affiliated Children's Hospital of Guangxi University of Science and Technology, Liuzhou, China; ^3^Guangxi Health Commission Key Laboratory of Birth Cohort Study in Pregnant Women of Advanced Age, Liuzhou, China

**Keywords:** patent ductus arteriosus, angiogenesis, growth factors, hsPDA, bronchopulmonary dysplasia (BPD), ibuprofen

## Abstract

**Introduction:**

Ibuprofen is one of the most common non-steroidal anti-inflammatory drugs used to close patent ductus arteriosus (PDA) in preterm infants. PDA is associated with bronchopulmonary dysplasia (BPD), while PDA closure by ibuprofen did not reduce the incidence of BPD or death. Previous studies have indicated an anti-angiogenesis effect of ibuprofen. This study investigated the change of angiogenic factors after ibuprofen treatment in preterm infants.

**Methods:**

Preterm infants with hemodynamically significant PDA (hsPDA) were included. After confirmed hsPDA by color doppler ultrasonography within 1 week after birth, infants received oral ibuprofen for three continuous days. Paired plasma before and after the ibuprofen treatment was collected and measured by ELISA to determine the concentrations of platelet-derived growth factor-BB (PDGF-BB) and vascular endothelial growth factor A (VEGF-A), and hypoxia-inducible factor-2α (HIF-2α).

**Results:**

17 paired plasma from infants with hsPDA were collected. The concentration of PDGF-BB and VEGF-A significantly decreased after ibuprofen treatment (1,908 vs. 442 pg/mL for PDGF-BB, 379 vs. 174 pg/mL for VEGF-A). HIF-2α level showed a tendency to decrease after ibuprofen treatment, although the reduction was not statistically significant (*p* = 0.077).

**Conclusion:**

This study demonstrated decreased vascular growth factors after ibuprofen exposure in hsPDA infants.

## Introduction

With the increasing survival of extremely preterm infants, the incidence of severe morbidities in survivors attracted significant attention (1–4). In these tiny infants, the natural closure rate of patent ductus arteriosus (PDA) is only 10–20% in the early of life (5, 6). PDA persisting in preterm infants is more prone to hsPDA, closely related to preterm infant outcomes, including death (7). An open ductus significantly influences the blood pressure and the pulmonary flow, leading to respiratory deterioration, including bronchopulmonary dysplasia (BPD) (2, 8). Theoretically, closure of PDA would reduce the incidence of BPD; however, this assumption was not supported by clinical evidence (9–11).

Ibuprofen is the most common medication for hsPDA closure and was broadly studied (5, 12–14). In our previous clinical study, we observed that ibuprofen successfully closed PDA but resulted in a higher incidence of BPD compared with naturally closed PDA cases (15). This finding was supported by a network meta-analysis, where the authors showed that ibuprofen increased the risk of BPD by 30% compared to indomethacin or placebo (16). By striking contrast, a recent randomized clinical trial comparing the effect of ibuprofen treatment and non-intervention in PDA management found that ibuprofen did not significantly influence the incidence of BPD (11). These contradictory findings call for experimental evidence on the effect of ibuprofen on postnatal lung development.

Previous experimental evidence has shown that ibuprofen inhibited angiogenesis in cultured human umbilical vein endothelial cells (17), neonatal rat ocular (18), and zebrafish embryonic development (19). Angiogenesis is recognized as an essential counterpart in the pathophysiology of BPD (20–22). However, whether ibuprofen increases the risk of BPD *via* inhibition of angiogenesis in preterm infants is still uncertain. Therefore, the purpose of this study was to explore the effect of ibuprofen exposure on angiogenesis-related growth factors in paired blood samples taken from preterm infants before and after ibuprofen treatment.

## Methods

### Study design and patients

**Figure 1:**
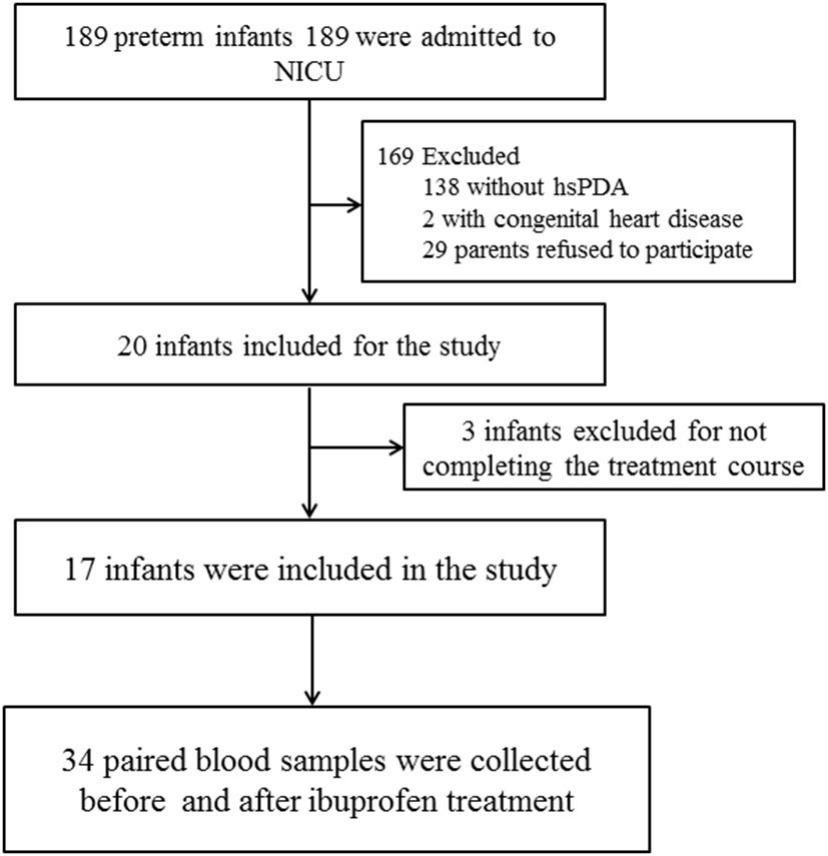
Flow chart of cases selection.

This prospective observational study was conducted at the neonatal intensive care unit (NICU) of Liuzhou Maternal & Child Healthcare Hospital between May and November 2021. All neonates born before 37 weeks gestation at our center were eligible for this study. We enrolled preterm infants with hemodynamically significant PDA (hsPDA) who received a standard ibuprofen regimen for the first time. Infants with severe appearance malformation, cyanotic congenital heart disease, and uncompleted ibuprofen treatment were excluded (Figure 1). Paired blood samples were collected before and after the ibuprofen treatment within 24 h. Clinical data of the infants, including demographic characteristics and outcomes, were collected. This study protocol was approved by the Medical Ethics Committee of Liuzhou Maternity & Child Healthcare Hospital[LMCHH (2021)-009]. We received written informed and consent for all participants from their parents or guardians.

### Echocardiography diagnosis and treatment

Color Doppler ultrasonography screening was routinely performed in all preterm infants who required respiratory support on the third day of life. Hemodynamically significant PDA (hsPDA) was diagnosed when a DA was present with predominantly left-to-right shunting across the DA associated with (1) left-atrium-to-aorta ratio (LA./Ao ratio) ≥ 1.5 and (2) PDA diameter (measured at its narrowest part, the end of pulmonary arterial) ≥ 1.5 mm, and when there were two out of the following clinical symptoms caused by PDA: (1) systolic or continuous murmur; (2) precordial pulsation distress; (3) hypotension; (4) respiratory distress; and (5) pulmonary edema or cardiomegaly (cardiothoracic ratio > 60%) on chest X-ray (23, 24). Echocardiograms were performed by an experienced pediatric sonographer using a Mindray M9 ultrasound scanner (Mindray, China) with a 10-MHz probe. Infants diagnosed with hsPDA received oral ibuprofen of 10 mg/kg on the first day, followed by two doses of 5 mg/kg every 24 h (25).

### Outcomes or adverse events

The outcomes of ibuprofen treatment were successful PDA closure, death, and BPD (defined as oxygen use for at least 28 days) or moderate-severe BPD (defined as oxygen use at a postmenstrual age of 36 weeks or discharge) (26). The adverse events included necrotizing enterocolitis (NEC), retinopathy of prematurity (ROP), and intraventricular hemorrhage (IVH) (any grade based on the Papile criteria) (27).

### Laboratory analyses of PDGF-BB, VEGF-A, and HIF−2α

Paired blood samples were collected with an EDTA tube before the first and after the last dose of ibuprofen. The samples were centrifuged for plasma at 4,000 rpm for 10 min within 2 h after collection, then frozen at −80 degree until analysis. PDGF-BB, VEGF-A, and HIF−2α concentrations were measured using commercial ELISA kits (# BMS2071, # BMS277-2 from Thermo Fisher, USA, and # ab227898 from Abcam, USA).

### Statistics

Continuous variables expressed as median and interquartile range (IQR) and categorical variables expressed as frequency and percentage were analyzed using GraphPad Prism 8.0. A paired *t*-test (non-parametric) was used for statistical analysis. A *p* ≤ 0.05 was considered statistically significant.

## Results

### Demographic and clinical characteristics

Thirty-four samples from 17 infants were collected (Figure 1). The demographic and clinical characteristics of the infants are shown in Table 1. The infants were born with a median gestational age of 29.5 weeks and a median birth weight of 1,300 grams.

**Table 1:** Demographic and clinical characteristics of 17 infants.

**Variables**	***n*** = **17**
Antenatal steroid treatment, *n* (%)	12 (70.6)
Preterm premature rupture of membranes, *n* (%)	3 (17.6)
Male, *n* (%)	9 (52.9)
GA at birth, median (IQR), wk	29.5 (26.6, 31.5)
Birth weight, median (IQR), gr	1,300 (970, 1,750)
Small for gestational age (SGA), *n* (%)	1 (5.9)
PDA diameter before treatment, median (IQR), mm	2.3 (1.7, 3.1)
Postnatal day of first blood sampling, median (IQR), day	6.0 (3.0, 6.5)
**Respiratory support before blood sampling**	
Invasive ventilation, *n* (%)	9 (52.9)
Non-invasive ventilation, *n* (%)	5 (29.4)
FiO_2_%, median (IQR)	0.30 (0.25, 0.35)
Vasoactive, *n* (%)	7 (41.2)
Blood product transfusion, *n* (%)	6 (35.3)

IQR, interquartile range; GA, gestational age; PDA, patent ductus arteriosus.

### Plasma PDGF-BB, VEGF-A, and HIF-2α levels significantly decreased after ibuprofen treatment

**Figure 2:**
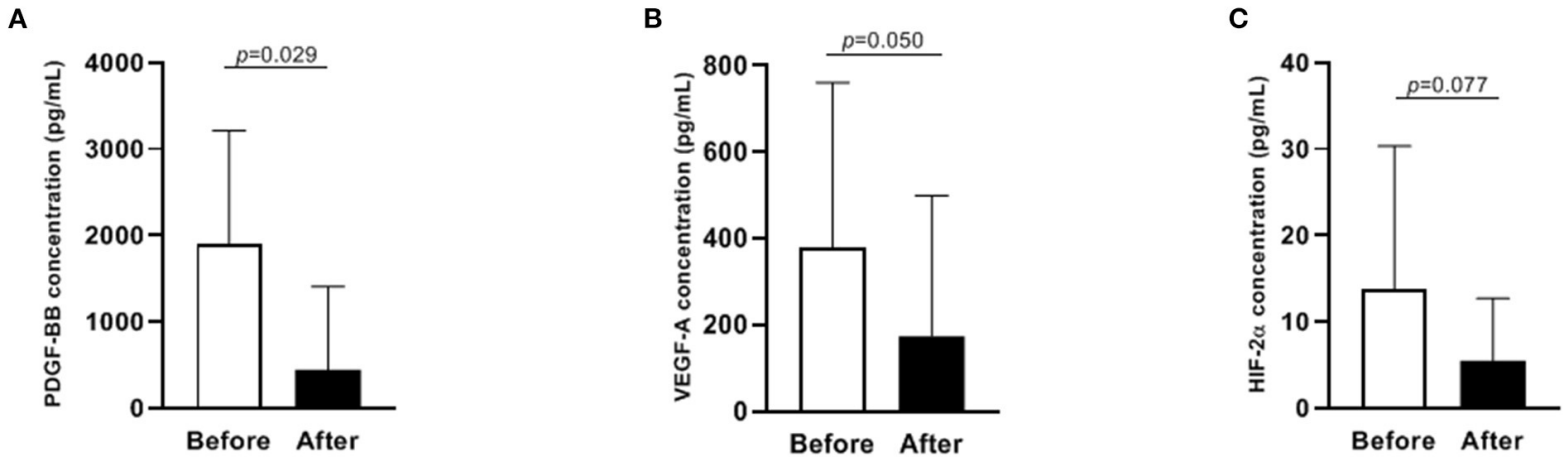
Levels of PDGF-BB, VEGF-A and HIF-2α before and after ibuprofen treatment in 17 infants **(A–C)**. Data were presented as median with IQR. PDGF-BB, platelet-derived growth factor BB; VEGF-A, vascular endothelial growth factor A; HIF-2α, hypoxia-inducible factor 2α. Only 12 paired data were shown in **(C)** because the concentration of HIF-2α was below the detection limit in the rest samples.

**Figure 3:**
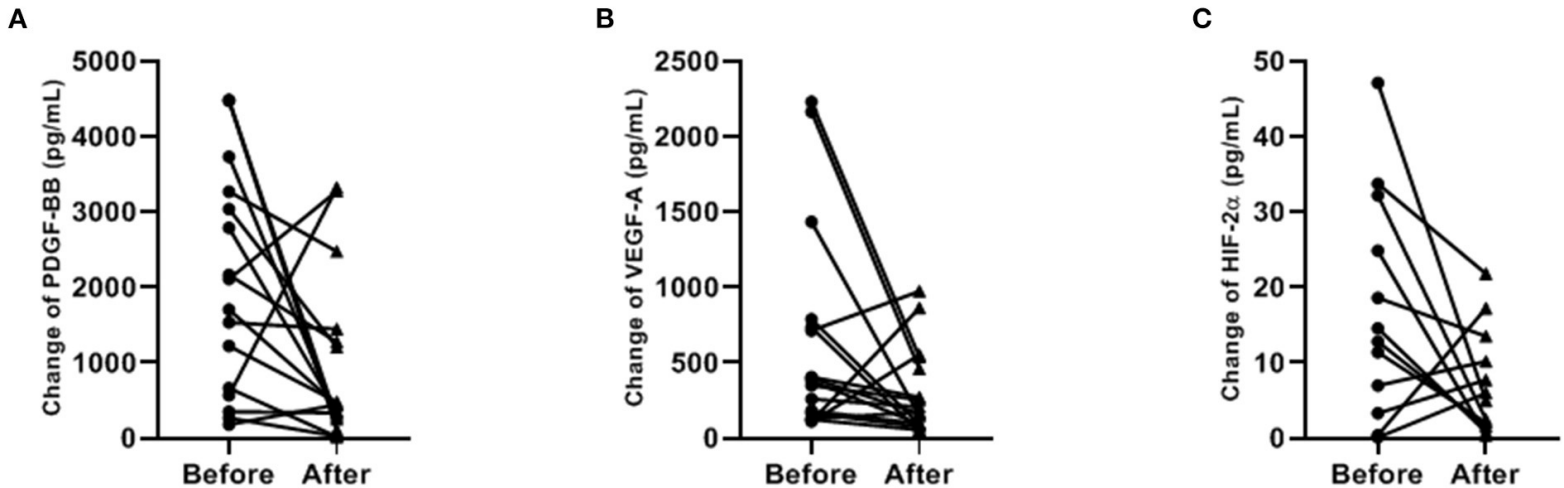
Paired dot plotting of antigenic factors levels before and after ibuprofen exposure **(A–C)**. PDGF-BB, platelet-derived growth factor BB; VEGF-A, vascular endothelial growth factor A; HIF-2α, hypoxia-inducible factor 2α. Only 12 paired data were shown in **(C)** because the concentration of HIF-2α was below the detection limit in the rest samples.

Concentrations of PDGF-BB and VEGF-A significantly decreased after ibuprofen exposure, from 1908 to 442 pg/mL for PDGF-BB (*p* = 0.029), and 379–174 pg/mL for VEGF-A (*p* = 0.050, Figure 2). The level of HIF-2α showed a tendency to warding decrease after ibuprofen treatment, although the decrease was not significant (*p* = 0.077, Figure 2). The before and after dot plots of different growth factors from all individuals were demonstrated in Figure 3. Furthermore, the changes in these angiogenic factors were not influenced by PDA status after ibuprofen treatment (Table 2).

**Table 2:** Concentrations of angiogenic factors stratified by PDA status after ibuprofen treatment.

**Angiogenic factors, pg/mL median (IQR)**	**PDA open**	**PDA close**	***P*** **-value**
PDGB-BB change	791.3 (−1,166.8, 1,833.1)	646.1 (−10.6, 2,348.5)	0.828
VEGF-A change	94.7 (−260.4, 695.8)	90.4 (11.7, 993.7)	0.770
HIF-2α change	9.3 (−4.4, 11.9)	13.8 (−0.4, 27.1)	0.465

PDA, patent ductus arteriosus; IQR, interquartile range. Change, defined as before minus after in the same infant.

### Neonatal outcomes of 17 infants

After ibuprofen treatment, the closure rate of PDA was 58.8% (10/17, Table 3). The rate of BPD was 70.6% (12/17), defined as oxygen use at the postnatal age of 28 days, or 29.4% (5/17) for moderate-severe BPD, defined as supplemental oxygen needed at postmenstrual age (PMA) 36 weeks or discharge. No hospital death was observed in these infants. The average hospital stay of these infants was 55.2 days. The rate of NEC, ROP, and IVH are shown in Table 3.

**Table 3:** Neonatal outcomes of 17 infants.

**Outcomes**	***N*** = **17**
PDA closure after treatment, *n* (%)	10 (58.8)
Death, *n* (%)	0 (0)
Bronchopulmonary dysplasia (BPD), *n* (%)	12 (70.6)
Moderate-severe BPD, *n* (%)	5 (29.4)
Necrotizing enterocolitis, NEC ≥ 2 stage, *n* (%)	1 (5.9)
Retinopathy of prematurity (ROP), *n* (%)	6 (35.3)
Intraventricular hemorrhage (IVH), *n* (%)	7 (41.2)
Duration of hospitalization, median (IQR), days	52.0 (33.0, 79.5)

PDA, patent ductus arteriosus; IQR, interquartile range.

## Discussion

Our experimental results show the inhibition of PDGF-BB, VEGF-A, and HIF-2α after ibuprofen treatment in premature infants. Considering the fundamental role of angiogenesis in the development of BPD, these findings could potentially explain the clinical observation that ibuprofen increases the risk of BPD and PAH shown in our and other studies (15, 16, 28, 29). To our best knowledge, this is the first study to show the concentration change of angiogenesis-related growth factors after ibuprofen exposure, in paired blood samples from premature infants.

Knowledge of the essential role of angiogenesis in BPD pathogenesis has now been broadly recognized (20, 30), as well as the role of angiogenic factors (31). The development and growth of blood vessels are regulated by various angiogenic factors (32), including but not strict to PDGF-BB, VEGF-A, and HIF-2α. In normal vascular growth, PDGF-BB is released from endothelial cells and binds to PDGFR-b expressed on the surface of emerging mural cells (33–35). Unlike PDGF-AA, confirmed to be involved in alveolarization through interaction with its receptor PDGFR-α (36), PDGF-BB receives less attention from researchers. PDGF-BB deficiency mice showed aberrant embryonic blood vessel formation, probably caused by reduced smooth muscle cells and pericytes (34). A previous study showed that two isoforms of PDGF receptors (PDGFR-α and -β) both reduced in either BPD infants or BPD mice (37). Similarly, Arjaans et al. found that a lower PDGF-BB level was associated with a higher risk for BPD in premature infants (28). Although the causality between decreased PDGF-BB and BPD has not been confirmed yet, we speculate that the decrease in PDGF-BB might be responsible for the increased risk for BPD after ibuprofen exposure.

Similarly, VEGF and HIF are both potent angiogenic factors, playing an essential role in vascular development (20, 32, 38). Previous studies found reduced VEGF levels in bronchoalveolar lavage fluid (BALF) from BPD infants (39), and VEGF receptor inhibition blunted both angiogenesis and alveolarization in the neonatal murine lung (40). Intriguingly, overexpression of VEGF-A did not rescue lung development but resulted in capillary leakage (41). This evidence suggests that precise regulation of VEGF is needed to ensure normal lung development. HIF is an upstream regulator of VEGF (42). Similar to VEGF, HIF-2α expression decreased in BPD mice, and inhibition of HIF-1α and HIF-2α induced a BPD phenotype (43). To be more specific, airway administration of HIF-1α preserved postnatal alveolarization in newborn mice exposed to hyperoxia without affecting HIF-2α expression (43), suggesting a more predominant role of HIF-1α in alveolarization.

We have demonstrated a decrease in PDGF-BB, VEGF-A, and HIF-2α after ibuprofen treatment in premature infants in this study. Because infants without ibuprofen were not included as controls, we could not confidently exclude a physiologically decrease in angiogenic factors studied. According to Hellgren et al., both the VEGF-A and PDGF-BB concentrations increased in the first weeks after birth (44), while both parameters dropped dramatically in the current study. Besides, these remarkable decreases occurred within 3–4 days. One would not expect such an abrupt change in a relatively short period. Furthermore, by comparing the changes in each angiogenic factor between infants with PDA closure and PDA open after ibuprofen treatment, we found no significant difference between the two groups, indicating these changes were PDA status independent. Taken together, we infer that the decreases in angiogenic factors in this study were attributed to ibuprofen exposure. This hypothesis is supported by a previous study reporting that ibuprofen inhibited secretion of angiogenic factors basic fibroblast growth factor (bFGF) and VEGF in HMEC-1 cells under inflammatory conditions (45).

There were some limitations in our study. The sample size was small due to limited parents signing the informed consent form. Furthermore, the average gestational age of the included infants was 29.5 weeks, indicating they are not the exact high-risk population for BPD. Moreover, confounding factors interfering with the expression of angiogenic factors such as mechanical ventilation, hyperoxia, and inflammation, were not studied.

We have demonstrated an anti-angiogenic effect of ibuprofen in preterm infants, explaining the increased risk of BPD after the ibuprofen treatment found in clinical studies. These findings might alert clinicians on PDA treatment with ibuprofen in premature infants, especially in prolonged and repeated courses.

## Data availability statement

The raw data supporting the conclusions of this article will be made available by the authors, without undue reservation.

## Ethics statement

The studies involving human participants were reviewed and approved by the Medical Ethics Committee of Liuzhou Maternity; Child Healthcare Hospital. Written informed consent to participate in this study was provided by the participants' legal guardian/next of kin. Written informed consent was obtained from the individual (s), and minor (s)' legal guardian/next of kin, for the publication of any potentially identifiable images or data included in this article.

## Author contributions

XC and CY contributed to the research design. XH, YW, and DH contributed to the acquisition and analysis of the data. XH and XC drafted the manuscript. XH, DH, YW, BL, DZ, YZ, BW, ZH, XC, and CY contributed to the collection and interpretation of the data. All authors critically reviewed and revised the manuscript and approved the final manuscript.

## Funding

This study was supported by Guangdong Basic and Applied Basic Research Foundation (2020B1515120034 to CY), Shenzhen Science and Technology Program (JCYJ20210324131006018 to CY), the institutional project (FYA2018018 to CY), and Key Research and Development Program of Guangxi (No. Guike AB18126056).

## Conflict of interest

The authors declare that the research was conducted in the absence of any commercial or financial relationships that could be construed as a potential conflict of interest.

## Publisher's note

All claims expressed in this article are solely those of the authors and do not necessarily represent those of their affiliated organizations, or those of the publisher, the editors and the reviewers. Any product that may be evaluated in this article, or claim that may be made by its manufacturer, is not guaranteed or endorsed by the publisher.
